# Quantifying the impact of optical surface guidance in the treatment of cancers of the head and neck

**DOI:** 10.1002/acm2.12867

**Published:** 2020-04-06

**Authors:** Wenbo Wei, Pericles J. Ioannides, Varun Sehgal, Parima Daroui

**Affiliations:** ^1^ Department of Radiation Oncology University of California Irvine Orange CA USA; ^2^ Department of Radiation Oncology The Permanente Medical Group Santa Clara CA USA

**Keywords:** head and neck cancer, patient setup, radiation therapy, surface guided radiation therapy (SGRT)

## Abstract

Surface guided radiation therapy (SGRT) is increasingly being adopted for use in radiation treatment delivery for Head and Neck (H&N) cancer patients. This study investigated the improvement of patient setup accuracy and reduction of setup time for SGRT compared to a conventional setup. A total of 60 H&N cancer patients were retrospectively included. Patients were categorized into three groups: oral cavity, oropharynx and nasopharynx/sinonasal sites with 20 patients in each group. They were further separated into two (2) subgroups, depending on whether they were set up with the aid of SGRT. The Align‐RT™ system was used for SGRT in this work. Positioning was confirmed by daily kV‐kV imaging in conjunction with weekly CBCT scans. Translational and rotational couch shifts along with patient setup times were recorded. Imaging setup time, which was defined as the elapsed time from the acquisition of the first image set to the end of the last image set, was recorded. Average translational shifts were larger in the non‐SGRT group. Vertical shifts showed the most significant reduction in the SGRT group for both oropharynx and oral cavity groups. Pitch corrections were significantly higher in the SGRT group for oropharynx patients and higher pitch corrections were also observed in the SGRT groups of oral cavity and nasopharynx/sinonasal patients. The average setup time when SGRT guidance was employed was shorter for all three treatment sites although this did not reach statistical significance. The largest time reduction between the SGRT and non‐SGRT groups was seen in the nasopharynx/sinonasal group. This study suggests that the use of SGRT decreases the magnitude of translational couch shifts during patient setup. However, the rotational corrections needed were generally higher with SGRT group. When SGRT was employed, a definite reduction in patient setup time was observed for nasopharynx/sinonasal and hypopharynx cancer patients.

## 1 Introduction

Accurate patient positioning during the radiation treatment of head and neck (HN) patients to replicate the setup of CT simulation is crucial since multiple Organs at Risk (OARs) such as parotid glands, larynx, and esophagus, etc. are in close proximity to the tumor. Setup errors could potentially result in significant underdose to the tumor and/or overdose to one or more OARs. Traditionally, patient positioning adjustments have been made by aligning surface marks on thermoplastic marks or marker tattoos placed at the time of CT simulation and then fine‐tuned by orthogonal kV imaging (kV‐kV), cone‐beam CT (CBCT), etc. However, due to the complexity of the anatomy within the head and neck area, the internal organs at risk are still subject to an average of 2–3 mm displacement with the immobilization devices after the initial setup^1^, ^2^. This not only introduces mechanical uncertainties but also could potentially lead to repeat imaging and consequently prolong the treatment setup time. Therefore, there is a clinical necessity for establishing a more comprehensive approach to improve the initial setup accuracy and limit the number of radiographs or in‐room scans needed to ensure accurate patient positioning.

Surface‐guided radiation treatment (SGRT) was designed to determine in real‐time the position of an object by tracking the positions of either active or passive infrared markers attached to the object.^3^, ^4^, ^5^ The SGRT system uses a projector to cast 3D pattern points onto the patient and the position of the points of reflection is determined using multiple cameras.^6^, ^7^ The primary advantage of this technique is that it is noninvasive and does not utilize ionizing radiation for image capture. Several recent publications have documented a benefit for various disease sites including left breast cancer,^8^, ^9^ brain cancer,^10^, ^11^ and lung cancer,^12^ The benefits come from two perspectives namely setup and monitoring. Quicker patient setup can potentially reduce the imaging dose while active patient monitoring can potentially enhance localization and treatment delivery accuracy. However, compared with other anatomical sites, only a limited number of studies have reported on patient setup utilizing SGRT for radiation therapy of the head and neck region (HN region).^13^, ^14^, ^15^, ^16^ Zhao et al.^15^ reported on a pilot trial to investigate the feasibility and setup accuracy of the minimal face and neck mask immobilization with optical surface guidance. They enrolled 20 patients undergoing standard of care IMRT treatment to the head and neck area and employed both optical guidance as well as daily CBCT to determine any resulting setup errors. Surveys were administered to assess patient comfort and total treatment time and resulting shifts were recorded. Another component of the study reported by Zhao et al.^15^ was to compare two shoulder restriction methods to determine which one provided better patient setup. They concluded that approximately 5–10% of the fractions had shifts greater than 5 mm and about 0–3% had shifts greater than 7 mm. The average total treatment time was determined to be about 20 min, which was in line with the time slot allocated for head and neck IMRT treatments. They also reported that patients gave high comfort scores to the open immobilization masks and that moldable cushions provided better patient setup than shoulder stirrups.

Wiant et al.^14^ compared the impact of open and closed thermoplastic masks on anxiety, claustrophobia, intrafraction motion and posture preservation in 50 patients undergoing radiation treatments to the head and neck region. The patients were prospectively randomized into open and closed mask groups and daily volumetric imaging (MVCT or CBCT) was used for all patients. Their results showed that only about 4% of the fractions had movements greater than 2 mm and no significant difference between the open and closed mask groups as far as posture analysis was concerned. Their results also showed that the open mask group reported lower mean values of anxiety and claustrophobia compared to the closed mask group but the differences were not statistically significant. Gopan and Wu^13^ examined the accuracy of surface imaging for rigid and non‐rigid setups in head and neck cancer radiotherapy by comparing internal 3D image pixel values for CT registration and surface spatial information for AlignRT registration. They concluded that while Align‐RT system could be used for verifying and correcting daily rigid setup for head and neck radiotherapy further investigations were needed to improve the accuracy for non‐rigid realignment. Li et. al. developed a new enlarged precut open‐face thermoplastic mask with eyes, nose, and mouth shown and the mask can achieve clinically acceptable levels of 1.0 ± 0.5 mm for both immobilization and surface imaging.^22^

The above‐mentioned studies established that employing SGRT resulted in a high level of accuracy for the fractionated treatment of head and neck cancers. These studies also showed that patient anxiety and claustrophobia levels were in general lower when an open mask used for SGRT replaced the conventional closed mask traditionally used for head and neck treatments. At least two of these studies also concluded that when SGRT was employed the treatment times were similar to those for non‐SGRT fractionated IMRT head and neck radiotherapy treatments. All these studies employed CT scans (MVCT, Helical CT, and CBCT) to establish the accuracy of SGRT for head and neck treatments. None of the above‐mentioned studies explored in detail the potential role of daily kV‐kV imaging in conjunction with weekly CBCT to establish the accuracy of SGRT treatments for head and neck radiotherapy. These studies also did not consider the potential reduction in the need of pre‐treatment imaging and patient setup time when SGRT guidance was employed for patient setup.

The primary objective of this study was to assess the improvement of patient setup accuracy and reduction of setup time when SGRT was employed compared to conventional non‐SGRT setup for head and neck radiotherapy using Volumetric Modulated Arc Therapy (VMAT) treatment delivery. A secondary objective was to determine if SGRT could benefit all the sub anatomical structures at different levels and depths within the HN region, such as oropharynx, oral cavity and nasopharynx/sinonasal. A tertiary objective was to assess the potential reduction in patient setup time when SGRT guidance was employed as compared to conventional patient setup methods.

## 2 Materials and methods

### 2.A Patient population

A total of 66 head and neck patients who underwent either definitive or post‐operative radiation therapy between 2014 and 2018 were retrospectively included in this study. Based on different treatment sites, the patients were initially categorized into four groups including oral cavity, oropharynx, nasopharynx/sinonasal, and hypopharynx/larynx groups. The analysis of the hypopharynx/larynx group failed to proceed due to an insufficient patient population with SGRT aided treatment at the time of analyses. Therefore, 60 patients remained in the study from the other three treatment site groups with 20 patients in each group. The patients were further separated into two (2) subgroups, depending on whether the patients were set up with the assistance of the surface imaging tracking system or purely with physical marks drawn on the open thermoplastic mask by the therapists. Ten patients were included in each subgroup.

### 2.B Clinical workflow

**Fig. 1:**
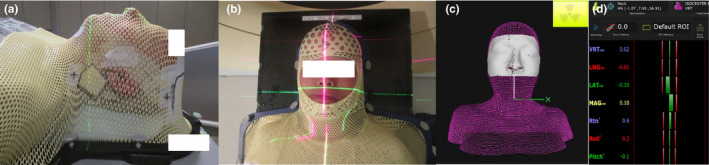
(a) Non‐surface guided radiation therapy (SGRT) setup: A conventional closed thermoplastic S‐frame mask molded on the patient to cover the head and shoulder regions. Marks are placed on the mask to align with the lasers for patient setup, (b) SGRT setup: An open S‐frame mask was used with the mid‐face opening and the ROI was set in the area for SGRT monitoring, (c) SGRT monitoring: the surface rendering, generated from the CT dataset in the SGRT system to assist with daily patient setup, (d) SGRT tolerance: SGRT patients were first set up with optical image guidance to ensure that the isocenter was within the 2 mm/2° tolerance. On‐board imaging (kV‐kV/cone‐beam CT) was subsequently performed to verify and correct patient alignment.

All patients were simulated on a Philips Brilliance Big Bore computed tomography (CT) scanner (Philips Medical Systems, Cleveland, OH, USA) and all treatment planning CT scans were acquired with 2 mm slice thickness. The widely used optical tracking system AlignRT (VisionRT, London, UK) was used in this study. It consists of three ceiling‐mounted stereoscopic camera pods in the treatment room. The system was calibrated monthly and verified daily, using a calibration plate per manufacturer’s recommendations as well as departmental quality assurance policies and procedures. The non‐SGRT group was treated in a conventional closed thermoplastic S frame mask (Q‐Fix, Integrated Shim™ for Portrait™ S‐Frame) to cover the head and shoulder regions (Fig. 1a), whereas the SGRT group was treated with an open S frame mask (Fig. 1b) attached to the S‐Frame. A standard head support was selected to fit the patient and minimize the gap under the neck.

Three CT radio‐opaque/metal (BB) markers were placed on the masks at the time of simulation to assist with treatment localization. On the patient’s first treatment fraction, after aligning the patient to the treatment isocenter using kV imaging, marks were drawn on the thermoplastic masks for the guidance of the remaining treatment fractions. For the SGRT patients, the target region of interest (ROI) was defined in the center of the opening in the S‐frame thermoplastic mask fabricated during CT simulation and the patient’s surface contour generated from the CT dataset was imported into the SGRT system. Similar to the approach used in the study by Zhao et al.,^15^ for subsequent treatment fractions, the surface rendering from the previous day was employed for the daily setup (Fig. 1c). Interfractional variation such as weight loss was tracked using weekly CBCT images. All HN radiation treatments were delivered on a Varian Truebeam (Varian Medical Systems, Palo Alto, CA, USA) with 2.5 mm high‐definition MLC. Standard head and neck cancer treatment dose fractionation regimens ranging from 60 to 70 Gy in 30–35 fractions were prescribed. All patients were treated with VMATand the number of arcs used ranged from 2 to 4 with a higher number of arcs employed for more complex cases. Daily kV‐kV imaging and weekly CBCT was performed in both the SGRT and non‐SGRT arms.

On the day of treatment, SGRT patients were first set up to surface marks and subsequently with optical image guidance to the reference parameters. Beam hold thresholds were set at 2 mm for translational shifts (longitudinal, lateral and vertical shifts) and 2° for rotational shifts (pitch, roll, and yaw) [Fig. 1(d)]. Positioning was verified either by daily paired orthogonal kV images (kV‐kV) or by weekly CBCT imaging. Image registration was based on bony landmarks for kV‐kV imaging and soft‐tissue registration for CBCT imaging. Post matching of kV‐kV/CBCT images, all shifts were applied using a six‐dimensional robotic couch (Varian PerfectPitch™, Varian Medical Systems, Palo Alto, CA, USA). Non‐SGRT patients were set up to the marks drawn on the thermoplastic masks followed by kV‐kV or CBCT imaging. Translational and rotational couch shifts (both kV and CBCT) along with patient setup time for the SGRT and non‐SGRT patients were recorded for every treatment fraction. For those cases where the patient received more than one set of paired kV images, the couch adjustments after the first acquired images were used since they typically represent the largest shifts and subsequent adjustments were used for fine‐tuning.

### 2.C Data analysis

Data for this study were obtained through Aria Version 13.6 record and verify database (Varian Medical Systems, Palo Alto, CA, USA) using International Classification of Diseases (10th Edition), codes for nasopharynx (11.0–11.9), sinonasal (31.0–31.9), oral cavity (2.0–6.9), hypopharynx (12.0–13.9) and larynx (32.0–32.9). Six degrees of freedom couch adjustments (translational and rotational) after on‐board imaging were compared between the SGRT group and non‐SGRT group. Systematic error, the standard deviation of the individual patient’s mean couch position for SGRT patients, was also calculated.^17^ Vectors of translational shifts were determined by using the root of sum of squares of the three directional shifts. For each patient, the percentage of treatment fractions receiving >5 mm, >7 mm and >10 mm vector shifts were recorded. The average percentage of fractions that received various vector changes were compared for non‐SGRT and SGRT groups in each patient category. Imaging setup time, which was defined as the elapsed time from the acquisition of the first image set to the end of the last image set, was recorded for every treatment fraction and compared between the two groups. Statistical analysis was performed using the two‐tailed t‐test using Microsoft Excel 2016 (Microsoft Corporation, Redmond, WA, USA). A p‐value <0.05 was considered statistically significant.

## 3 Results

### 3.A Translational shift

**Fig. 2:**
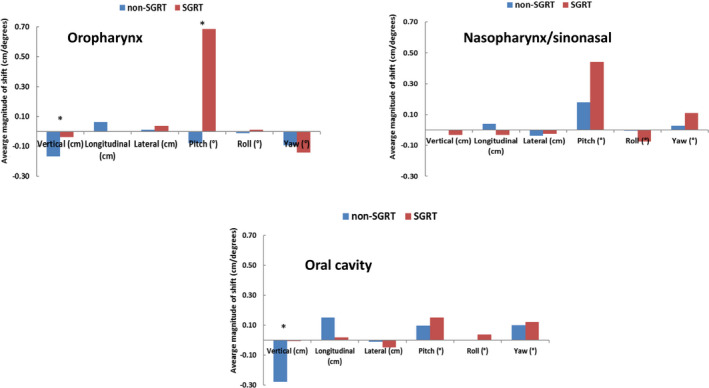
Translational and rotational corrections in non‐surface guided radiation therapy (SGRT) and SGRT groups using combined (kV‐kV + CBCT) imaging data. Translational shifts improved in the oropharynx and oral cavity SGRT groups with vertical shifts showing the most significant difference. Rotational corrections degraded in all three treatment sites when SGRT was utilized. Pitch adjustment was highest in the oropharynx group. Asterisks denote statistically significant differences.

Comparing the setup shifts of two groups, the average translational shifts were generally larger in the non‐SGRT group (Fig. 2). Out of the three translational corrections, vertical shifts showed the most significant reduction in the SGRT group for both oropharynx (−1.7 ± 1.1 mm for a non‐SGRT group vs. −0.04 ± 0.08 mm for SGRT group, p = 0.01) and oral cavity groups (−2.8 ± 1.3 mm for a non‐SGRT group vs. −0.1 ± 0.12 mm for SGRT group, p < 0.01). The longitudinal correction showed a trend of reduced magnitude also in the oropharynx and oral cavity groups with the greatest difference observed in the oral cavity group (1.5 ± 1.5 mm for a non‐SGRT group vs. 0.2 ± 0.9 mm for SGRT group, p = 0.08). The lateral couch corrections were comparable between SGRT and non‐SGRT groups for all three treatment sites. The mean translational and rotational shifts for the three groups are listed in Table 1.

**Table 1:** Summary of mean setup shifts and systematic error per treatment site in the non‐SGRT and SGRT groups (combined kV‐kV/CBCT data).

			Vertical (mm)	Longitudinal (mm)	Lateral (mm)	Pitch (°)*	Roll (°)*	Yaw (°)*
Oropharynx	Non‐SGRT	Mean	−1.68	0.63	0.09	−0.08	0.26	−0.09
		Systematic error	1.09	1.52	0.95	0.95	1.07	0.34
	SGRT	Mean	−0.38	−0.03	0.36	0.68	0.20	−0.14
		Systematic error	0.79	0.86	1.22	0.49	1.21	0.40
		*P* value	0.010	0.277	0.618	0.051	0.912	0.784
Nasopharynx/sinonasal	Non‐SGRT	Mean	−0.04	0.39	−0.38	0.18	0.01	0.03
		Systematic error	1.40	1.26	1.90	0.52	0.93	0.35
	SGRT	Mean	−0.32	−0.33	−0.25	0.44	−0.45	0.11
		Systematic error	0.58	0.54	0.63	0.66	1.06	0.31
		*P* value	0.585	0.138	0.857	0.420	0.409	0.656
Oral cavity	Non‐SGRT	Mean	−2.77	1.50	−0.11	0.10	−0.33	0.10
		Systematic error	1.33	1.46	1.82	0.65	0.90	0.19
	SGRT	Mean	−0.06	0.19	−0.49	0.15	0.29	0.12
		Systematic error	1.16	0.89	1.11	0.42	0.81	0.44
		*P* value	0.000	0.078	0.596	0.835	0.156	0.891

SGRT, surface guided radiation therapy; CBCT, cone‐beam CT.

* Pitch, Yaw data from kV‐kV images and Pitch, Yaw and Roll data from CBCT images.

### 3.B Rotational shift

On the other hand, the overall kV‐kV rotational corrections (pitch and yaw) displayed different patterns for different correction categories (Fig. 2). Pitch corrections were significantly higher in the SGRT group for oropharynx patients (−0.08° ± 0.95° for a non‐SGRT group vs. 0.68° ± 0.49° for SGRT group, p < 0.05) and higher pitch corrections were also observed in the SGRT groups for oral cavity and nasopharynx/sinonasal patients although no significant difference was shown in these two groups (p = 0.84 and 0.42, respectively). The yaw corrections were also relatively higher in the SGRT groups.

### 3.C Systematic error

**Fig. 3:**
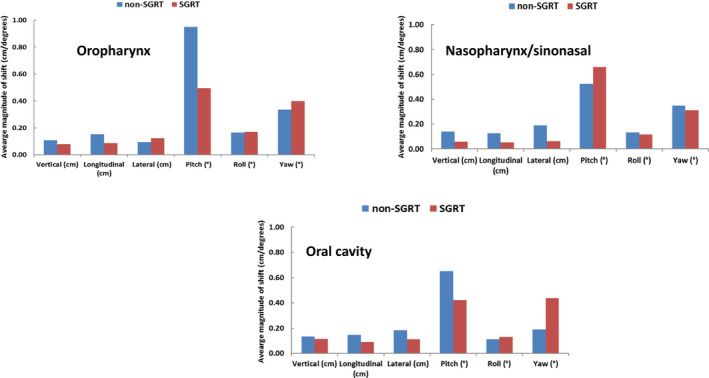
Systematic errors were reduced in general for the non‐ surface guided radiation therapy group with combined (kV‐kV + CBCT) imaging. A similar pattern was observed for all three treatment sites.

Systematic errors were generally higher for the non‐SGRT group (Fig. 3). Overall, systematic error on translational shifts was 1.4 mm for the non‐SGRT group vs. 0.9 mm for the SGRT group, while the minimal difference was seen on the average systematic error of rotational corrections (0.38° for SGRT vs. 0.35° for non‐SGRT). With respect to the individual patient population, the systematic errors showed a similar pattern among the three groups. For the translational shift, the nasopharynx/sinonasal group showed the greatest improvement in systematic error (1.4 mm without SGRT vs. 0.6 mm with SGRT in a vertical direction, 1.3 mm vs. 0.5 mm in a longitudinal direction and 1.9 mm vs. 0.6 mm in a lateral direction). Regarding rotational corrections, the systematic error for the pitch showed the most variability among different treatment sites. The SGRT group showed lower systematic errors for the oropharynx (0.95° without SGRT vs. 0.49° with SGRT) and oral cavity (0.65° vs. 0.42°), and higher systematic error for nasopharynx/sinonasal (0.52° vs. 0.66°).

### 3.D CBCT

**Fig. 4:**
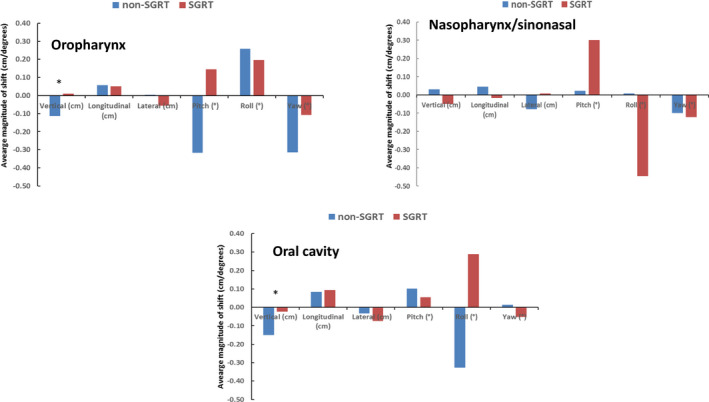
Translational and rotational corrections in non‐surface guided radiation therapy (SGRT) and SGRT groups with cone‐beam CT imaging only. As observed with kV‐kV imaging, translational shifts showed the most significant decrease in the oropharynx and oral cavity groups with SGRT while the rotational corrections were inferior with SGRT. Asterisks denote statistically significant differences.

**Fig. 5:**
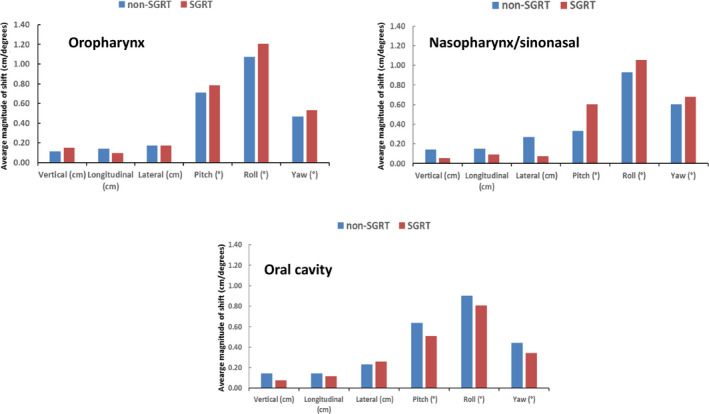
Systematic errors were similar in general for the two groups when only cone‐beam CT data were analyzed. The most improvement was observed in translational shifts of nasopharynx/sinonasal patients with surface guided radiation therapy.

Furthermore, we separately analyzed the CBCT data and two hundred sixty‐six (266) CBCT scans were extracted from SGRT and non‐SGRT patients for setup shift comparison. The results are summarized in Table 2. Similar to previous combined (kV‐kV + CBCT) results, SGRT showed the most improvement in the vertical shift with oropharynx patients having a mean shift of −1.1 mm without SGRT and 0.1 mm with SGRT (p = 0.03), and oral cavity patients having a shift of −1.5 mm (non‐SGRT) and −0.2 mm (SGRT) (p = 0.03) (Fig. 4). Longitudinal and lateral shifts were comparable and moderately improved with SGRT although no significant difference was observed. For the rotational shifts, SGRT, in general, showed worse alignment, which was consistent with the results obtained with combined (kV‐kV + CBCT) data. Less systematic error on average was seen in CBCT translational setup after implementing SGRT (1.7 mm non‐SGRT vs. 1.2 mm SGRT) (Fig. 5). Similar to the previous combined (kV‐kV + CBCT) data, the nasopharynx/sinonasal group showed the most decrease in translational systematic error (1.4 mm without SGRT vs. 0.6 mm with SGRT in a vertical direction, 1.5 mm vs. 0.9 mm in a longitudinal direction and 2.7 mm vs. 0.7 mm in a lateral direction). Rotational errors (pitch, roll, and yaw) were in general similar between the two groups.

**Table 2:** Summary of average setup shifts and systematic error per treatment site in the non‐SGRT and SGRT groups when only CBCT imaging was analyzed.

			Vertical (mm)	Longitudinal (mm)	Lateral (mm)	Pitch (°)	Roll (°)	Yaw (°)
Oropharynx	non‐SGRT	Mean	−1.12	0.57	0.04	−0.32	0.26	−0.31
		Systematic Error	1.16	1.42	1.75	0.71	1.07	0.47
	SGRT	Mean	0.10	0.52	−0.56	0.14	0.20	−0.11
		Systematic Error	1.49	0.97	1.74	0.79	1.21	0.53
		p value	0.034	0.927	0.474	0.208	0.912	0.393
Nasopharynx/sinonasal	non‐SGRT	Mean	0.32	0.44	−0.78	0.02	0.01	−0.10
		Systematic Error	1.40	1.49	2.70	0.33	0.93	0.60
	SGRT	Mean	−0.49	−0.16	0.07	0.30	−0.45	−0.12
		Systematic Error	0.57	0.90	0.73	0.60	1.06	0.68
		p value	0.188	0.367	0.442	0.259	0.409	0.949
Oral cavity	non‐SGRT	Mean	−1.50	0.84	−0.34	0.10	−0.33	0.01
		Systematic Error	1.43	1.44	2.32	0.64	0.90	0.44
	SGRT	Mean	−0.23	0.93	−0.74	0.05	0.29	−0.05
		Systematic Error	0.79	1.15	2.59	0.51	0.81	0.34
		p value	0.035	0.945	0.718	0.861	0.156	0.792

SGRT, surface guided radiation therapy; CBCT, cone‐beam CT.

### 3.E Percentage of fractions that received various vector changes

**Fig. 6:**
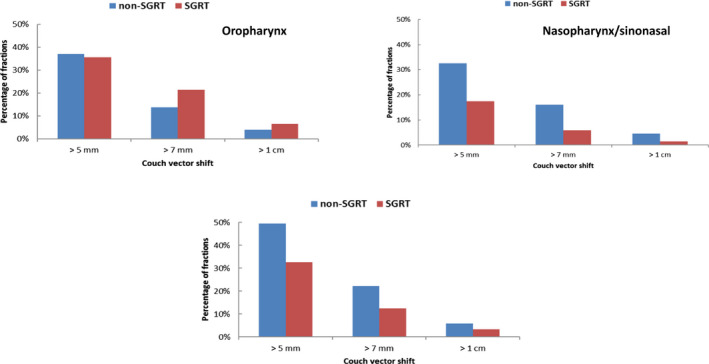
Percentage of fractions that received greater vector changes (> 5 mm,> 7 mm and> 10 mm). Both nasopharynx/sinonasal and oral cavity treatments had a higher percentage of fractions that received greater vector change without using surface guided radiation therapy.

Oral cavity patients were observed to have larger vector shifts in the most fractions, with on average 41% of fractions requiring a vector shift >5 mm, 17% fractions requiring a shift >7 mm and 5% fractions requiring a shift >10 mm. When comparing non‐SGRT and SGRT groups, both nasopharynx/sinonasal and oral cavity patients were observed to have a higher percentage of fractions requiring >5 mm, >7 mm and >10 mm vector shifts for the non‐SGRT group (Fig. 6). Oral cavity patients without SGRT recorded significantly more fractions with vector shifts greater than 5 mm (50% for non‐SGRT and 33% for the SGRT).

### 3.F Average setup time with onboard imager (kV and CBCT Imaging)

**Fig. 7:**
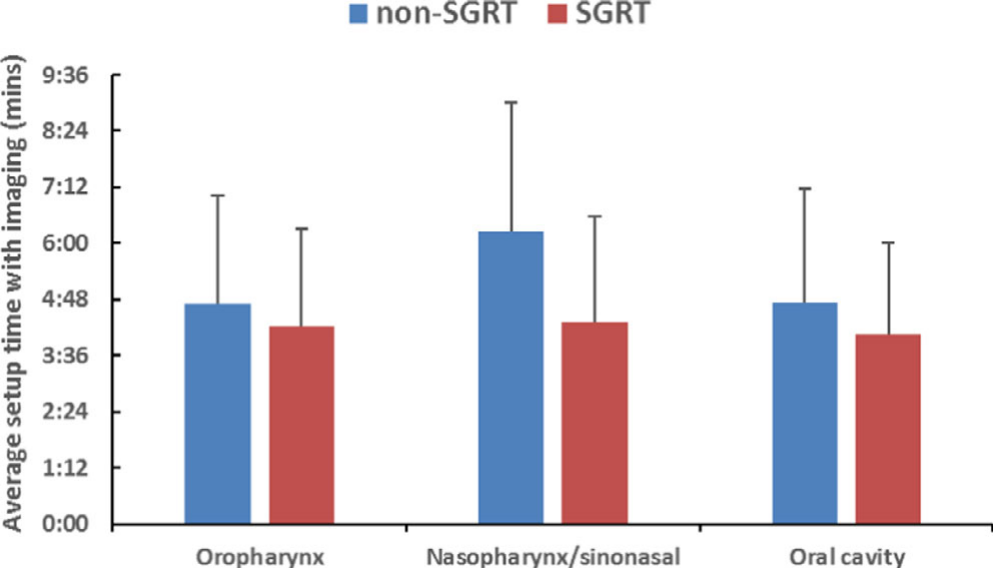
Average imaging setup time with and without surface guided radiation therapy.

The average setup time when both on‐board imaging and SGRT guidance were employed was shorter for all three treatment sites although no significant difference was observed (Fig. 7). The largest time reduction between the SGRT and non‐SGRT groups was seen in the nasopharynx/sinonasal group (6:14 ± 2:44 min vs 4:18 ± 2:16 min, p = 0.09).

## 4 Discussion

Since the application of SGRT in radiation therapy, a multitude of clinical data has been published on various anatomical sites. Deep inspiration breath‐hold (DIBH) treatments for breast cancer radiotherapy, using SGRT, reduce the doses to surrounding OARs, such as the heart, left anterior descending coronary artery and ipsilateral lung.^18^, ^19^, ^20^, ^21^ SGRT for treating intracranial metastases can produce clinical outcomes comparable to those for conventional frame‐based radiosurgery while providing greater patient comfort with an open‐faced mask and shorter treatment times.^14^, ^22^, ^23^ However, limited data is available in the literature for assessing the efficacy of SGRT for the HN radiation treatment setup. Because SGRT was designed to trace the patient’s surface motion, it was not clear whether SGRT could act as a good indicator for the anatomy sitting relatively deeper in the body such as the HN region compared to the breast tissue where the targets for tracking are more superficial. Another probable reason that the HN region may be more difficult to monitor is due to its inherent flexibility. SGRT is ideal for guidance in intracranial cases since the cranium is a rigid structure with limited motion relative to the facial surface.

The accuracy of patient setup using daily kV imaging coupled with weekly CBCT and comparing the open‐face and the conventional mask has not yet been presented in the literature. Compared with previous studies using SGRT for HN radiation treatment, our study design is different in that it focusses on assessing different treatment sites within the HN region including nasopharynx/sinonasal, oropharynx, and oral cavity. Due to varying anatomical depth and level for these three treatment sites in the HN region, different outcomes such as translational and rotational corrections were expected. In addition, we focused especially on the treatment setup with six‐dimensional couch shift and imaging preparation time. Our data are for the most part in line with the findings of the study by Zhao et. al. who assessed the feasibility and setup accuracy of minimal face and neck mask immobilization with SGRT and compared the setup shifts based on CBCT between the patients treated with shoulder cushion and shoulder retractors.^15^ Our data show similar results with the vertical and pitch corrections accounting as the largest shifts in translational and rotational corrections respectively. While the primary objective of the study by Zhao et al. was to confirm the feasibility of minimal coverage immobilization our study goes a couple of steps further and assesses the impact of employing SGRT for localizing patients being treated for head and neck cancer.

In this study, it has been shown that SGRT combined with hand‐placed marks as initial setup reference could lead to more accurate positioning. The average translational shifts were reduced with SGRT, especially the vertical shift. This indicates that the 3D matrix projected on the patient could potentially provide better guidance compared to the three physical marks on the thermoplastic immobilization mask. Utilization of SGRT has shown to have the most impact on the vertical shift. That may be because it is inherently difficult to mark the isocenter accurately on the thermoplastic mask so as to exactly align with the actual treatment isocenter. Since the mark was only placed on the patient’s mask on day one, it would only reflect the initial isocenter location. Patient’s anatomy may also be changing due to tumor response or weight loss during the course of radiation therapy. The physical marks would not be able to capture the change accordingly. On the contrary, when SGRT was utilized the reference image for every fraction was recorded post alignment with kV‐kV imaging and used as the baseline for the next day’s treatment. Thus employing the SGRT workflow could potentially limit the impact of the change in patient’s anatomy on the treatment setup. Surprisingly, the rotational corrections were increased with SGRT with the pitch being the most distinctive. This could be attributed to the rigid registration algorithm employed by the SGRT system.^5^, ^24^ The HN anatomical region is not truly a rigid body due to the presence of flexible bony structures.^25^ It may produce internal rotations that are different from the reference positions. Thus, the rotational changes might not be accurately represented by the anterior skin surface, which SGRT uses for registration. Therefore, it is not recommended that SGRT alone be used for localization of a non‐rigid patient setup.

Comparing different parameters within each treatment site groups, we determined that each group benefitted from SGRT application in one way or another. Oropharynx and oral cavity setups showed the greatest improvement in the translational couch shift. Systematic error showed a decreasing pattern in all three treatment groups with SGRT. The percentage of fractions in which the patients needed relatively larger shifts after the initial setup decreased with the utilization of SGRT for the nasopharynx/sinonasal and oral cavity groups. The rotational error increased in all three treatment sites but to a different extent(s). For example, the maximum vertical shift was −3.7 mm in the oropharynx group and decreased to −2.0 mm after implementing SGRT. The maximum yaw was −5.9°, which increased marginally to −7.9° after SGRT was employed.

The patient setup time using image guidance, a parameter of paramount importance as far as clinical resources is concerned, showed a decreasing trend when SGRT was utilized for nasopharynx/sinonasal patients. Utilization of SGRT results in a shortening of the overall treatment time, thus reducing potential patient discomfort and maintaining effectiveness. Also, limiting the imaging time decreases imaging radiation exposure.

One of the limitations of this study was that only one ROI was selected for SGRT tracking. The mid‐face area, which is typically selected as the ROI, is closer to the level of the nasopharynx region, but it is more distal from the oropharynx and oral cavity regions. Thus, anatomically it might not be the ideal area to place the ROI. Using thermoplastic masks that have an additional opening in the neck region could allow a second ROI to be tracked and thus potentially compensate for the discrepancy caused by the different anatomic levels. Another limitation is the small number of patients treated to the hypopharynx, which precluded any meaningful analysis of this patient group. Since the hypopharynx sits inferiorly to the other three anatomical regions, it would be interesting to determine if SGRT with the mid‐face ROI still improved patient localization.

## 5 Conclusions

In this study, we have presented data comparing treatment setup and pre‐treatment imaging time with and without SGRT in the H&N patients. Our results suggest that the use of SGRT decreases the magnitude and systematic errors of translational couch shifts during the patient setup, especially for oropharynx and oral cavity patients. Thus, it potentially improves setup accuracy by decreasing couch positioning uncertainty. However, the rotational corrections needed were generally higher with SGRT group, which suggests SGRT is best utilized in tandem with onboard radiographic imaging. When SGRT was employed, a definite time reduction in patient setup time was observed for nasopharynx/sinonasal cancer patients.

## Conflict of interests

The authors declare no conflict of interest.
